# The Effect of Adding Ready-to-Use Supplementary Food to a General Food Distribution on Child Nutritional Status and Morbidity: A Cluster-Randomized Controlled Trial

**DOI:** 10.1371/journal.pmed.1001313

**Published:** 2012-09-18

**Authors:** Lieven Huybregts, Freddy Houngbé, Cécile Salpéteur, Rebecca Brown, Dominique Roberfroid, Myriam Ait-Aissa, Patrick Kolsteren

**Affiliations:** 1 Department of Food Safety and Food Quality, Ghent University, Ghent, Belgium; 2 Action Contre la Faim, Paris, France; 3 Child Health and Nutrition Unit, Department of Public Health, Institute of Tropical Medicine of Antwerp, Antwerp, Belgium; London School of Hygiene & Tropical Medicine, United Kingdom

## Abstract

**Background:**

Recently, operational organizations active in child nutrition in developing countries have suggested that blanket feeding strategies be adopted to enable the prevention of child wasting. A new range of nutritional supplements is now available, with claims that they can prevent wasting in populations at risk of periodic food shortages. Evidence is lacking as to the effectiveness of such preventive interventions. This study examined the effect of a ready-to-use supplementary food (RUSF) on the prevention of wasting in 6- to 36-mo-old children within the framework of a general food distribution program.

**Methods and Findings:**

We conducted a two-arm cluster-randomized controlled pragmatic intervention study in a sample of 1,038 children aged 6 to 36 mo in the city of Abeche, Chad. Both arms were included in a general food distribution program providing staple foods. The intervention group was given a daily 46 g of RUSF for 4 mo. Anthropometric measurements and morbidity were recorded monthly. Adding RUSF to a package of monthly household food rations for households containing a child assigned to the intervention group did not result in a reduction in cumulative incidence of wasting (incidence risk ratio: 0.86; 95% CI: 0.67, 1.11; *p* = 0.25). However, the intervention group had a modestly higher gain in height-for-age (+0.03 Z-score/mo; 95% CI: 0.01, 0.04; *p*<0.001). In addition, children in the intervention group had a significantly higher hemoglobin concentration at the end of the study than children in the control group (+3.8 g/l; 95% CI: 0.6, 7.0; *p* = 0.02), thereby reducing the odds of anemia (odds ratio: 0.52; 95% CI: 0.34, 0.82; *p* = 0.004). Adding RUSF also resulted in a significantly lower risk of self-reported diarrhea (−29.3%; 95% CI: 20.5, 37.2; *p*<0.001) and fever episodes (−22.5%; 95% CI: 14.0, 30.2; *p*<0.001). Limitations of this study include that the projected sample size was not fully attained and that significantly fewer children from the control group were present at follow-up sessions.

**Conclusions:**

Providing RUSF as part of a general food distribution resulted in improvements in hemoglobin status and small improvements in linear growth, accompanied by an apparent reduction in morbidity.

**Trial registration:**

ClinicalTrials.gov NCT01154595

*Please see later in the article for the Editors' Summary.*

## Abstract 2

Lieven Huybregts and colleagues investigate how supplementing a general food distribution with a fortified lipid-based spread during a seasonal hunger gap in Chad affects anthropometric and morbidity outcomes for children aged 6 to 36 months.

## Editors' Summary

**Background:**

Good nutrition during childhood is essential for health and survival. Undernourished children are more susceptible to infections and are more likely to die from common ailments such as diarrhea than well-nourished children. Globally, undernutrition contributes to about a third of deaths among children under five years old. Experts use three physical measurements to determine whether a child is undernourished. An “underweight” child has a low weight for his or her age and gender when compared to the World Health Organization Child Growth Standards, which chart the growth of a reference population. A “stunted” child has a low height for his or her age; stunting indicates chronic undernutrition. A “wasted” child has a low weight for his or her height; wasting indicates acute undernutrition and can be caused by disasters or seasonal food shortages. Recent estimates indicate that about a fifth of young children in developing countries are underweight, and one third are stunted; in south Asia and west/central Africa, more than one tenth of children are wasted, a condition that markedly increases the risk of death.

**Why Was This Study Done?:**

In emergency situations, international organizations support affected populations by providing “general food distributions.” Recently, there have been claims that the provision of targeted nutritional supplements within a general food distribution framework effectively prevents child wasting, but there is little evidence to support these claims. In this cluster-randomized controlled trial, the researchers investigate the effect of a targeted daily dose of a “ready-to-use supplementary food” (RUSF; a lipid-based nutrient supplement) on indicators of undernutrition in 6- to 36-month-old, non-wasted children in Chad, a country beset by a severe food crisis. Political instability in this central African country has severely reduced the nutritional status of children, and annual droughts, which affect crop production, cause a “hunger gap” between June and October. In a recent survey, one fifth of children in Chad were wasted at the beginning of this hunger gap. A cluster-randomized trial randomly assigns groups of people to receive alternative interventions and compares the outcomes in the differently treated “clusters.”

**What Did the Researchers Do and Find?:**

The researchers randomly assigned fourteen household clusters in the city of Abeche, Chad, to the trial's intervention or control arm. All the households received a general food distribution that included staple foods; eligible children in the intervention households were also given a daily RUSF ration between June and September 2010. The researchers regularly measured the children's weights and heights, recorded illnesses reported by caregivers, and measured each child's blood hemoglobin level before and after the intervention to assess their risk of anemia, an indicator of poor nutrition. The addition of RUSF to the household food rations did not significantly reduce the cumulative incidence of wasting. That is, although fewer children in the intervention group became wasted during the trial than in the control group, this difference was not statistically significant—it could have happened by chance. However, compared to the children in the control group, those in the intervention group had a significantly greater gain in height-for-age (equivalent to a difference in height gain of 0.09 cm/month), slightly higher hemoglobin levels at the end of the study, which significantly reduced their anemia risk, and a significantly lower risk of self-reported diarrhea and fever.

**What Do These Findings Mean?:**

Although targeted RUSF provided as part of a general food distribution had no significant effect on wasting in young children in Abeche, Chad, the intervention improved their hemoglobin status and linear growth, and reduced illness among them. Why didn't targeted RUSF prevent wasting effectively in this trial? Maybe the effect of RUSF was diluted out by the effect of the general food distribution or maybe the trial was too short to see a clear effect. Most importantly, though, the trial may have been too small to see a clear effect—the researchers were unable to enroll as many children into their trial as they had planned because of political instability in Chad, and this probably limited the trial's ability to detect small differences between the control and intervention groups. Nevertheless, because these findings provide no clear evidence that adding RUSF to a household food ration effectively prevents wasting, alternative ways to prevent acute malnutrition in Chad and other vulnerable regions of the world should be investigated.

**Additional Information:**

Please access these websites via the online version of this summary at http://dx.doi.org/10.1371/journal.pmed.1001313.

- This study is further discussed in a *PLOS Medicine* Perspective by Kathryn Dewey and Mary Arimond
- Action Contra la Faim–France has a web page that describes the situation in Chad
- The United Nations Childrens Fund, which protects the rights of children and young people around the world, provides detailed statistics on child undernutrition; it has detailed information, including videos, about the current food crisis in Chad and the Sahel
- The WHO Child Growth Standards are available (in several languages)
- The United Nations provides information on ongoing world efforts to reduce hunger and child mortality
- The World Food Programme is the world's largest humanitarian agency fighting hunger worldwide; its website provides detailed information about malnutrition in Chad, including a video of the current food crisis in the country
- Starved for Attention is an international multimedia campaign launched in 2010 by Médecins Sans Frontiéres (MSF) and the VII Photo agency to rewrite the story of childhood malnutrition; information about MSFs work in Chad to tackle malnutrition is available

## Introduction

Acute malnutrition, or wasting, affects large numbers of children in developing countries. An analysis based on data from 139 countries found that the percentage of deaths attributable to wasting is about 10.2%. Wasted children have a three times higher risk of death compared to well-nourished children [1],[2]. Wasting adversely impacts the development of learning capacity and undermines future economic potential of both individuals and countries [2],[3].

Fortified lipid-based spreads, containing different concentrations of micronutrients, are broadly categorized as lipid-based nutrient supplements (LNSs). Several formulations of LNSs exist, including ready-to-use therapeutic food (RUTFs) and ready-to-use supplementary foods (RUSFs). RUTF has proven effective in the nutritional rehabilitation of children with severe acute malnutrition [4]–[8], leading the World Health Organization (WHO) to recommend the use of RUTF within the framework of community-based management of severe acute malnutrition without medical complications [8]–[10].

Based on this success, both RUTF and RUSF have been used in targeted supplementation to complement the dietary intake of moderately malnourished children or children at risk of malnutrition [11],[12]. Recent studies have assessed the effectiveness of provision of RUTF and RUSF in preventing malnutrition in non-wasted children in settings of permanent or seasonal food insecurity [13]–[15]. However, questions have been raised as to whether such practices are universally effective and cost-effective [16].

In emergency settings, “general food distributions” are implemented by international organizations to provide basic food support to affected populations. While there is a clear need for such interventions, it remains unclear whether such programs are effective in preventing child wasting, as rations are targeted at the household level. In addition, it has been suggested that targeting nutrition interventions to prevent child undernutrition might be more effective than curative treatment to reduce child undernutrition [17].

To date, we are aware of no study assessing the effectiveness of including a targeted nutritional supplement within a general household food distribution in order to address child malnutrition. We hypothesized that including a daily dose of 46 g of LNS as RUSF, for consumption by children between 6 and 36 mo of age, as part of a household food distribution program would reduce cumulative wasting incidence during the seasonal hunger gap (June to October). The study was designed as a two-arm cluster-randomized intervention study. Because the intervention could not be blinded to participants, we randomized by clusters rather than by individuals to avoid contamination (RUSF sharing between households) as much as possible (Text S2).

## Methods

### Study Area

Chad is a landlocked country of the Sahelian belt, located in central Africa. Agricultural practices are dependent on natural rainfall, with staple crops of millet and sorghum harvested once per year. The country suffers from annual droughts affecting cereal production. During the agricultural periods of 2009/2010, poor rain and pest attacks had a serious impact on cereal production, resulting in a decrease in production of around 34% compared to the previous five years' average [18].

This study was conducted in the city of Abeche, the capital city of the Ouaddaï region in eastern Chad. Since 2006, this part of the country has been characterized by chronic political instability; this situation has led to the degradation of nutritional status in children under 5 y of age. Recently, the non-governmental organization Action Contre la Faim–France (ACF-France) conducted two cross-sectional nutritional surveys in representative samples of the population of children under 5 y in Abeche. A first survey of 853 children was conducted at the beginning of the rainy season (June 2009) and revealed a prevalence of wasting of 20.6%, with 3.2% severe wasting, using the National Center for Health Statistics (NCHS) growth reference [19]. A second survey involving 650 children under 5 y was carried out during the post-harvest period (January 2010). This survey reported a wasting prevalence of 16.8%, with 2% severe wasting [20]. This prevalence is above the WHO threshold of 15%, indicating the need for national and international intervention [21].

A supplementary feeding program, admitting moderately and severely wasted children without medical complications, was operational in six health centers in the city. Each year, these centers experienced an increase in the number of children admitted during the annual hunger gap period (June to October), as a consequence of the deterioration in household food security.

### Study Sample and Randomization

**Figure 1:**
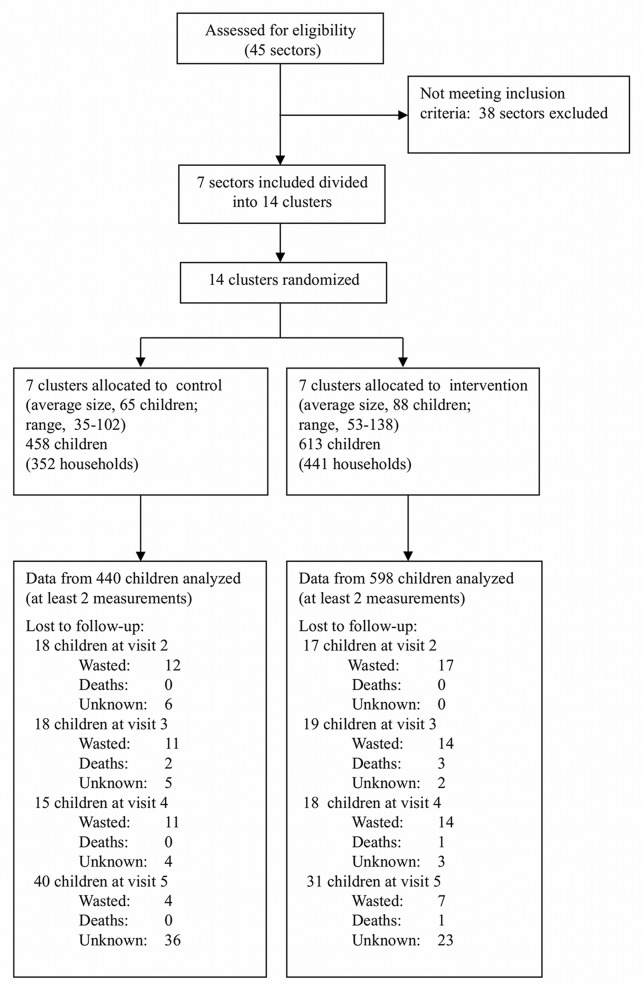
Study flow chart.

The study was conducted in seven vulnerable sectors of the city of Abeche that were preselected from a total of 45 administrative sectors. These seven sectors were identified based on data from a community network organized by ACF-France involving a set of socioeconomic (housing, electricity, access to education and health facilities), sanitary (recurrent floods, presence of dumps, access to clean water), and nutritional (proportion of children admitted to ACF-France nutrition rehabilitation programs) criteria [22]. The selected sectors were subdivided into 14 geographical clusters using main roads and rivers as cluster boundaries. The cluster was the unit of randomization; random assignment was conducted through an official ceremonial gathering with officials and community members. Fourteen papers with cluster numbers were drawn blindly from a bag. Drawn clusters were alternatively assigned to the intervention group and control group. This draw resulted in seven clusters assigned to the intervention group and seven to the control group (Figure 1).

We designed the study as a two-arm cluster-randomized controlled pragmatic trial targeting children from 6 to 36 mo of age from vulnerable households. A household was considered to be “vulnerable” when it met one of the following criteria: (1) household head being disabled, pregnant, or lactating, or (2) an economic dependency ratio of 4∶1 or more (number of economically inactive versus active household members).

The study's target group was children between 6 and 36 mo old. The inclusion criteria for the study were being non-wasted (weight-for-height ≥80% of the NCHS reference median, and lack of bilateral pitting edema) and being from a “vulnerable” household, as described above. Although a more recent WHO international growth reference exists [23], the study used the NCHS growth reference to conform with the Chad national protocol for the management of malnutrition [24].

In order to detect a 50% reduction in the cumulative incidence of wasting over a period of 4 mo, with a statistical power of 80%, a type I error of 5%, an estimated mean cluster size of 100 children, and an intra-cluster correlation of 0.01, a sample size of 1,220 children was calculated to be needed. Taking into account a study dropout of 15%, a total sample size of 1,435 children was projected.

### Intervention

The study was initially designed to be part of a conditional food-for-training program, with caretakers receiving a monthly food ration in exchange for participation in a sensitization program organized by ACF-France. However, at the inception of the study, this conditional character of the food distribution was omitted because the security situation worsened considerably. As a consequence, this meant that attendance at the scheduled sessions was lower than expected.

Households of both arms received a monthly food package representing a daily ration of 425 g of sorghum (*Sorghum bicolor*), 25 g of legumes (*Lens culinaris*), 25 g of bleached palm oil, 20 g of sugar, and 5 g of iodized salt, all provided by the World Food Programme. This ration was estimated to cover approximately 86% (≈1,800 kcal) of the daily energy requirements for a population at risk of an emergency [25]. The number of food rations distributed per household was proportional to its size: 1.5 rations for households of one to two members, three rations for households of three to four members, five rations for households of five to six members, eight rations for households of seven to nine members, and 11 rations for households of ten or more members.

Children from the intervention group received a monthly quantity of RUSF (Plumpy'Doz, Nutriset) representing a daily ration of 46 g (≈247 kcal/d). Recommendations on dosage and frequency of consumption by targeted children were made to caretakers and repeated at each follow-up visit. The intervention lasted 4 mo (June 2010 to September 2010). The composition of the RUSF is given in Table 1.

**Table 1:** Composition of a daily dose (46 g) of RUSF.

Component	Amount
Weight of daily ration, g	46
Energy, kcal	247
Protein, g	5.9
Lipids, g	16
Linoleic acid, g	2
α-Linolenic acid, g	0.3
Vitamin A, µg	400
Vitamin E, mg	6
Thiamin, mg	0.5
Niacin, mg	6
Pantothenic acid, mg	2
Vitamin B-6, mg	0.5
Folic acid, µg	160
Vitamin B-12, µg	0.9
Vitamin C, mg	30
Magnesium, mg	60
Zinc, mg	4
Iron, mg	9
Copper, mg	0.3
Potassium, mg	310
Calcium, mg	387
Phosphorus, mg	275
Selenium, µg	17
Manganese, mg	0.17
Iodine, µg	90

Contrary to the international recommendation [25], we did not include a fortified food (e.g., fortified corn-soy blend) in the food rations, to avoid the risk of a micronutrients overdose. Indeed, the intervention RUSF already provided the recommended nutrient intake for most of the micronutrients, and it was judged logistically infeasible to organize two different food rations, i.e., one with (control) and one without (intervention) additional multiple micronutrients (MMNs).

Prior to this intervention, an acceptability test was conducted in a convenience sample of 30 non-wasted children. Caretakers were given a single dose of 46 g and were asked to feed their child ad libitum during 20 min. Subsequently, the remaining portion was weighed, and the consumed quantity calculated by subtraction. This test revealed that on average 75.8% (95% CI: 65.6, 85.9) of the offered quantity was consumed. As proposed by Adu-Afarwuah et al. [26], we considered 50% consumption of the offered quantity to be meaningful on such short duration. Since the lower end of the 95% confidence interval is greater than 50% of the amount of RUSF offered to the children, the supplement was considered acceptable.

We encouraged the mothers to bring their children with them to the monthly food distributions, regardless of their intervention status. Children were enrolled from early June to mid-July 2011 and scheduled to come for four follow-up visits. At each visit, child anthropometric measurements and morbidity were recorded. Children who were classified as moderately wasted (weight-for-height <80% of NCHS reference median) or severely wasted (weight-for-height <70% of NCHS reference median) were discharged from the study and referred to a community-based management of acute malnutrition program located within the city's nutrition rehabilitation centers. All participating mothers were given a family food ration as described above.

### Measurements

We used a pretested questionnaire to collect data on socioeconomic (education, occupation, and assets ownership) and demographic characteristics of enrolled households. Child age at screening was estimated using a locally adapted event calendar if a birth certificate was unavailable.

Anthropometric measurements were conducted monthly using a standard procedure and calibrated instruments. Weight was measured using an electronic SECA 835 scale. Wooden planks were made to ensure scales' stability and to allow measurement to the nearest 0.1 kg. Children were measured naked or with very light underwear, and without necklaces. Recumbent length (<24 mo) and height (≥24 mo) were recorded to the nearest 0.1 cm using a wooden measuring board (Menuiserie Besnard). Mid-upper arm circumference (MUAC) was measured using a non-stretchable tape (Octopuss). We recorded MUAC to the nearest 0.1 cm. Episodes of diarrhea, respiratory tract infection, and fever were recalled for 1 wk before the monthly interview. Respiratory tract infection was diagnosed through reports by the mother/caregiver of persistent cough or difficulty in breathing during the last week (yes/no). A diarrheal episode was defined as having at least three loose stools within a day. Fever episodes were diagnosed by mother/caretaker during the last week (yes/no). In case of death, we carried out a verbal autopsy adapted from WHO standards [27],[28]. Hemoglobin concentration was measured at baseline (June) and at the end of the intervention (November) or when a child was discharged from the study, using a daily calibrated electronic device HemoCue Hb 201+.

Standardized forms were used to collect data. Measurements were taken by teams of trained nurses and assistants. The accuracy and precision of the measurements was assessed during standardization sessions prior to the study [29]. Anthropometric data were recorded twice on separate forms by different field teams to avoid the second recording being influenced by the first. Data collectors were blinded to group assignment when measurements were taken.

### Data Analysis

The primary study outcome was the cumulative incidence of acute malnutrition or wasting defined as weight-for-height Z-score (WHZ) <−2 [23] or presence of bilateral pitting edema. Secondary outcomes included mean WHZ change over time, prevalence of stunting at end point defined as height-for-age Z-score (HAZ)<−2, mean HAZ change over time, MUAC change over time, mean hemoglobin concentration at end point, prevalence of anemia at end point (hemoglobin <110 g/l), and morbidity. WHO Z-scores were calculated in STATA 11.2 using the ZSCORE06 command [30]. Although the national nutrition protocol in Chad prescribed the use of the NCHS growth reference, we opted to analyze the data using the WHO international growth references to make our results more comparable to recent studies. It is noteworthy to mention that none of the study conclusions were altered when analyzing the outcome data using the NCHS growth reference. Children providing at least two measurements with complete covariate data were included for analysis. To assess a possible bias in missing outcome data, we reanalyzed the data after imputation of the missing outcome data using the last value carried forward method.

Proportions, means, and standard deviations (SD) were presented for the baseline variables. A principal component analysis was used to create a proxy index for household socioeconomic status (SES) based on the possession of various assets declared by a household. These asset variables were collected upon inclusion and were self-reported by participating mothers. They were coded as binary variables. The ones that showed relevant contribution (>15%) to the combined SES proxy score factor were selected [31]. The principal component analysis factor with the highest eigenvalue was considered a variable that sufficiently described the household SES. The factor scores were categorized into tertiles. The lowest 33.3% of households according to SES score were classified as poor, the highest 33.3% as rich, and the rest as average economic status.

All analyses were conducted on an intention-to-treat basis. The effect of the intervention on the continuous outcomes WHZ, weight, HAZ, length, and MUAC was analyzed using linear mixed models that included cluster, household, and child as random effects to account for clustered observations. A random slope between follow-up time and growth outcomes was fitted to account for the individual growth curve. For both WHZ and HAZ, it was found that the addition of a quadratic term (random effect) of follow-up time versus the outcome improved the fit of the model significantly using likelihood ratio testing. Fixed effects included in the model were child's sex, SES, and child's age at inclusion, and WHZ at inclusion and HAZ at inclusion, for the outcomes mean WHZ and mean HAZ, respectively. All covariates were centered around their grand mean.

We calculated mean incidence rates for both arms, with confidence intervals derived from a Poisson distribution adjusted for clustering. We examined the intervention effect on the cumulative incidence of wasting, anemia, and self-reported diarrhea, respiratory tract infection, and fever. Incidence rate ratios were calculated using generalized linear and latent mixed models (GLLAMM). The GLLAMM procedure in STATA 11.2 allows the fitting of Poisson regression models with random effects cluster, household, and child. We opted to analyze binary wasting data using Poisson regression modeling to be able to present an adjusted risk ratio rather than an odds ratio (OR), as recommended for prospective studies [32],[33]. A robust estimation of the variance was adopted to relax the assumption of a Poisson distribution for binary data, as was proposed by [34]. We analyzed the difference in end point stunting and anemia prevalence between arms using mixed-effects logistic regression analysis with random effects cluster and household. All models were adjusted for fixed effects: child's sex and age at inclusion, SES at inclusion, and baseline values of the outcome of interest. In addition, we adjusted the models with the outcome morbidity for the number of recalls that were recorded.

Given the small number of clusters, we included in all models interactions between fixed effects and exposure time to adjust for potential imbalances and to gain statistical efficiency. We also assessed whether a uniform dose of RUSF for children of all ages would have had the same effect. In doing so, we assessed whether child's age at study inclusion modified the effect of RUSF on primary and secondary outcomes. For this purpose, we included a triple interaction between age at inclusion (continuous variable), exposure time, and intervention in all models. The addition of this interaction term to the models was evaluated using a likelihood ratio test. It is important to add that the study was not powered for such an interaction, so the result remains exploratory.

All the data were double-entered in EpiData version 3.1 (EpiData Association) by two data clerks. Statistical analyses were conducted using STATA 11.2 (Statacorp). The statistical significance for all analyses was set at 5%, and all tests were two-sided.

### Ethical Issues, Study Registration, and Participant Safety

The study protocol was reviewed and approved by the Ethics Committee of the University Hospital of Ghent in Belgium. Because an official ethics committee does not exist in Chad, the study protocol was reviewed and approved at an ad hoc gathering of representatives of the National Nutrition and Food Technology Center, representatives of the Ministry of Health, and the Ministry of Non-Governmental Organization Affairs. The trial protocol is available as Text S1.

## Results

Overall, baseline characteristics were balanced between the intervention and control groups, although more children were included in the intervention group. Also, mean HAZ in the intervention group was slightly lower than in the control group (Table 2). The striking balance in gender between arms was due to chance. The overall sample size considered for analysis was 1,038 children in 784 households: 598 children in the intervention group, and 440 children in the control group. A post hoc calculation of the inter- and intra-cluster variance of incidence of wasting resulted in an intra-cluster correlation coefficient of 0.0075 (design effect = 1.55), whereas an intra-cluster correlation coefficient of 0.01 (design effect = 2) had been assumed in calculating the necessary sample size. From June to October 2010, 86 children were lost to follow-up (Figure 1). Based on the NCHS reference, 38 control and 52 intervention children were found wasted at follow-up visits and were referred to a treatment program for moderate malnutrition. About 44% of children were younger than 24 mo at enrollment, and 56% were aged 24 mo or older. The majority of the infants included were anemic at baseline, although the prevalence did not differ by arm. Children contributed 1,427 and 2,199 mo of follow-up in the control and intervention groups, respectively, representing 87.1% and 97.0% of the scheduled follow-ups (*p*<0.001). The mean number of monthly follow-up visits was 3.2 (SD 1.0) and 3.7 (SD 0.7) for children in the control and intervention groups, respectively.

**Table 2:** Child characteristics at inclusion by study arm.

Characteristic	Control Arm	Intervention Arm
**Number of clusters**	7	7
**Number of children, ** ***n*** ** (percent)**	440 (42.4)	598 (57.6)
**Child-time, months**	1,427	2,199
**Age, months**	24.2 (10.3)	23.6 (9.9)
**Age category, ** ***n*** ** (percent)**		
6–11 mo	78 (17.7)	104 (17.4)
12–17 mo	63 (14.3)	82 (13.7)
18–23 mo	51 (11.6)	77 (12.9)
24–29 mo	78 (17.7)	138 (23.1)
30–36 mo	170 (38.7)	197 (32.9)
**Sex, ** ***n*** ** (percent)**		
Male	220 (50)	299 (50)
Female	220 (50)	299 (50)
**Weight, kg**	9.6 (2.1)	9.6 (2.1)
**Height, cm**	79.9 (9.3)	80.1(9.4)
**MUAC, cm**	14.0 (1.0)	14.0 (1.0)
**WHZ**	−1.13 (0.76)	−1.12 (0.80)
**HAZ**	−1.85 (1.43)	−1.65 (1.56)
**Wasting, ** ***n*** ** (percent)**		
WHZ<−2	59 (13.4)	81 (13.6)
WHZ<−3	1 (0.2)	3 (0.5)
**Stunting, ** ***n*** ** (percent)**		
HAZ<−2	198 (45.0)	245 (41.0)
HAZ<−3	98 (22.3)	106 (17.7)
**Hemoglobin, g/l** a	104 (17)	104 (16)
**Anemia (hemoglobin <110 g/l)** a	270 (61.5)	370 (61.9)
**SES classification, ** ***n*** ** (percent)**		
Low	151 (34.3)	196 (32.8)
Average	148 (33.6)	198 (33.1)
High	141 (32.1)	204 (34.1)

Data are mean (SD), unless stated otherwise.

a Hemoglobin concentration was not measured in one child in the control group.

Compared to baseline values, mean WHZ for both arms was slightly higher at end point, while mean HAZ was slightly lower. We found no difference in the incidence of wasting (incidence rate ratio: 0.86; 95% CI: 0.67, 1.11; *p* = 0.25) or mean change in WHZ (−0.002 WHZ/mo; 95% CI: −0.032, 0.028; *p* = 0.89) between the arms. The difference in weight increase between arms was 0.02 kg/mo (95% CI: −0.01, 0.04; *p* = 0.10). Children in the intervention group had a significantly higher linear growth velocity of 0.03 HAZ-score/mo (95% CI: 0.02, 0.05; *p*<0.001) compared to the control group (Table 3). This observed difference was equivalent to a small difference in height gain of 0.09 cm/mo (95% CI: 0.04, 0.14; *p*<0.001). Identical ponderal growth in control and intervention group was confirmed by a lack of difference in MUAC growth. Age at inclusion was not found to modify the intervention effects on child anthropometric measurements. Observed intervention effects did not noticeably change when missing outcome data were imputed using the last value carried forward method (data not shown).

**Table 3:** Effects of preventive RUSF on child anthropometry.

Outcome	Control Arm (*n* = 440)	Intervention Arm (*n* = 598)	*p*–Value
**Wasting**			
End point mean WHZ (SD)	−1.09 (0.95)	−1.05 (0.93)	
Intervention effect (95% CI), Z-score/mo^a^	Reference	−0.002 (−0.032, 0.028)	0.89
Cumulative episodes WHZ<−2	174	241	
Number of observed child-months	1,427	2,199	
Number of episodes per child-month (95% CI)^b^	0.12 (0.10, 0.14)	0.11(0.09, 0.14)	
Incidence rate ratio (95% CI)^c^	Reference	0.86 (0.67, 1.11)	0.25
**Stunting**			
End point mean HAZ (SD)	−2.06 (1.39)	−1.79 (1.46)	
Intervention effect (95% CI), Z-score/mo^a^	Reference	0.03 (0.01, 0.04)	<0.001
End point prevalence of stunting, percent (*n*)	52.3 (230)	46.2 (276)	
OR of end point stunting (95% CI)^d^	Reference	0.69 (0.45, 1.07)	0.099
**MUAC**			
End point MUAC, cm (SD)	14.1 (1.2)	14.3 (1.1)	
Intervention effect (95% CI), cm/mo^a^	Reference	0.01 (−0.02, 0.04)	0.49

a Analyzed using a linear mixed model with random effects cluster, household, and child, adjusted for child's age at baseline, child's sex, SES, and baseline value.

b Confidence intervals are estimated from a Poisson model adjusted for clustering.

c Analyzed using a mixed Poisson regression model with random effects cluster, household, and child, adjusted for child's age at baseline, child's sex, SES, and baseline value.

d Analyzed using a mixed logistic model with random effects cluster and household, adjusted for child's age at baseline, child's sex, SES, and baseline value.

After adjustment for age, sex, SES, and morbidity status at inclusion, we found that children from the RUSF group had lower risk of self-reported diarrhea by 29.3% (95% CI: 20.5, 37.2; *p*<0.001) and fever by 22.5% (95% CI: 14.0, 30.2; *p*<0.001), compared to the control group (Table 4). Effects on morbidity did not appreciably change when missing data were imputed.

**Table 4:** Effect of preventive RUSF on child morbidity.

Outcome	Control Arm (*n* = 440)	Intervention Arm (*n* = 598)	*p*-Value
Number of child-months recalled^a^	333	513	
**Diarrhea**			
Number of episodes	388	416	
Number of episodes per child-month (95% CI)^b^	1.17 (0.98, 1.39)	0.81 (0.68, 0.97)	
Incidence rate ratio (95% CI)^c^	Reference	0.71 (0.63, 0.80)	<0.001
**Fever**			
Number of episodes	448	548	
Number of episodes per child-month (95% CI)^b^	1.35 (1.21, 1.50)	1.07 (0.98, 1.17)	
Incidence rate ratio (95% CI)^c^	Reference	0.77 (0.70, 0.86)	<0.001
**Respiratory tract infection**			
Number of episodes	231	328	
Number of episodes per child-month (95% CI)^b^	0.69 (0.51, 0.95)	0.64 (0.58, 071)	
Incidence rate ratio (95% CI)^c^	Reference	0.87 (0.76, 1.01)	0.07

a Calculated by number of recalls×recall duration.

b Confidence intervals are estimated from a Poisson model adjusted for clustering.

c Analyzed using a mixed Poisson regression model with random effects cluster, household, and child, adjusted for child's age at baseline, child's sex, SES, and morbidity status at baseline.

RUSF significantly increased mean hemoglobin concentration at end point by 3.8 g/l (95% CI: 0.6, 7.0; *p* = 0.02), resulting in significantly lower odds of anemia (OR: 0.52; 95% CI: 0.34, 0.82; *p* = 0.004) for children in the intervention group (Table 5).

**Table 5:** Effect of preventive RUSF on hemoglobin concentration and anemia at end point.

Outcome	Control Arm (*n* = 409)	Intervention Arm (*n* = 573)	*p*-Value
Hemoglobin concentration at end point (SD), g/l	102.5 (15.2)	105.8 (14.3)	
Intervention effect (95% CI), g/l^a^	Reference	3.8 (0.6, 7.0)	0.02
Number of anemic cases at end point (percent)	273 (66.7)	324 (56.5)	
OR of end point anemia^b^	Reference	0.52 (0.34, 0.82)	0.004

End point hemoglobin values were unavailable for 31 and 25 participants from the control and intervention groups, respectively.

a Analyzed using a linear mixed model with random effects cluster and household, adjusted for child's age at baseline, child's sex, SES, and hemoglobin concentration at baseline.

b Analyzed using a mixed logistic regression model with random effects cluster and household, adjusted for child's age at baseline, child's sex, SES, and anemic status at baseline.

For the analysis of the intervention effect on morbidity and hemoglobin status, we did not find evidence that age at study inclusion modified the reported intervention effects (results not shown).

## Discussion

Adding RUSF to a package of household food rations did not result in a reduction in wasting incidence. On the other hand, the RUSF intervention had positive effects on mean linear growth (as indicated by HAZ), and resulted in a lower incidence of diarrhea and fever symptoms. We also found a significant increase in hemoglobin concentration and a reduced risk of anemia in children who were given RUSF.

The absence of an effect on wasting incidence could have multiple explanations. First, the energy contribution of RUSF may have been “diluted” by the general food distribution, which mainly provided a supplement of energy and protein. We did not systematically monitor whether or not children received food rations. However, post-distribution monitoring surveys carried out 2 wk after each distribution reported that more than 70% of children under 5 y had three meals or more per day in both arms (ACF-France, unpublished data). Furthermore, a previous study noted that energy-dense spreads have little effect on children's habitual diet and breast milk intake [35],[36], although additional dietary assessment studies investigating the impact of RUSF on diet substitution are warranted. Second, the energy dose of 46 g (≈247 kcal) daily RUSF and the duration of the supplementation could have been insufficient to support ponderal growth, particularly for the older children in the cohort. However, no significant triple interactions were found between age at baseline, intervention, and follow-up time on wasting incidence and mean ponderal growth. Furthermore, it is possible that the RUSF may have been shared with other children in the household. However, if this were done to a large extent, we may not have seen the observed intervention effects on secondary outcomes like HAZ and hemoglobin concentration. Also, program staff emphasized during distribution sessions that RUSF was intended only for the targeted child and that the family food rations could serve to feed other children. Finally, caution should be exercised when interpreting the reported difference in wasting incidence between arms, as the study is clearly underpowered to assess such small differences.

Children receiving RUSF supplementation showed a modest, but statistically significant, increase in mean linear growth (as indicated by HAZ). This finding is in line with a program using a 6-mo supplementation with RUSF in 6- to 36-mo-old Nigerians [15]. Although this effect was small, it is noteworthy because the supplementation lasted only 4 mo. This finding is also consistent with other previous studies. A significant reduction in severe stunting was reported among 6- to 28-mo-old Malawians after a 12-mo intervention [37]. Significant length gains were reported among 6- to 36-mo-old Nigerians after a 6-mo intervention with RUTF [14], in 6- to 12-mo-old Ghanaians after a 6-mo intervention with the LNS Nutributter [38], in 6- to 17-mo-old underweight Malawian infants after 12 wk of supplementation with milk-based fortified spreads [39], and in 3- to 6-y-old Algerians after a 6-mo intervention with a spread fortified with vitamins and minerals [40].

The group receiving RUSF had a significantly lower risk of diarrhea and fever compared to the control group. These findings are not confirmed by previous studies using RUTF or RUSF, except in the study by Ciliberto et al., which found a lower prevalence of diarrhea, fever, and cough in a group of moderately malnourished children given RUTF compared to a group that received standard therapy [4].

Significant positive intervention effects were also found on mean hemoglobin concentration and the prevalence of anemia. A 46-g daily dose of RUSF provided not only an average of 9 mg of iron per day but also addressed deficiencies in folic acid, vitamin B12, vitamin A, and riboflavin, which are known to limit erythropoiesis [41]. Similar results were found for Nutributter and Nutritabs in Ghana [42] and for milk-based fortified spreads in Malawi [39]. Lopriore et al. also showed that supplementation with a nutrient-dense fat-based spread fortified with vitamins and minerals produced a 2-fold higher increase in hemoglobin concentration after 6 mo of intervention compared to the 6-mo difference in a control group of 3- to 6-y-old children [40].

The absence of an effect on ponderal growth, but modest effects on morbidity, linear growth, and, most of all, hemoglobin could suggest that a MMN effect is at play. For example, zinc, one of the micronutrients added to RUSF, is currently recommended as adjunct therapy by the United Nations Children's Fund and WHO for the treatment of diarrhea [43]. A number of studies support its therapeutic and prophylactic role [44]–[48]. In addition, two systematic reviews concluded that MMN supplementation in young children improved micronutrient status and had modest effects on linear growth [49],[50]. Golden proposed that providing empty calories to children with MMN deficiencies would lead to ponderal growth without any effect on linear growth [50]. The author argues that MMNs, along with protein and energy, are essential building blocks necessary to make skeletal tissue to sustain linear growth. In that respect, we could regard MMNs as essential cofactors for linear growth, responsible for the observed effect on growth [51].

These observations could lead to the speculative hypothesis that supplementation with MMN supplements like powders or tablets might result in the same effects as RUSF if basic food rations were provided. Very few studies have compared RUSF to other MMN formulations to support child nutritional status. An interesting study by Adu-Afarwuah et al. compared three MMN supplements: Sprinkles, crushable Nutritabs, and 20 g of Nutributter. In addition to greater weight gain, children in the Nutributter group also experienced a greater gain in length compared to children given Nutritabs (*p*<0.05) and Sprinkles (*p*>0.05) [38].

One important additional benefit that lipid-based nutrient supplements offer is the lipid component. In addition to providing a small amount of essential fatty acids, which hold a potential to support child growth [52], the lipid component serves as an essential matrix that ensures that fat-soluble vitamins like vitamin A, D, and E are properly absorbed [53]. Particularly when the child's diet is poor in fat, this would provide leverage to increase the efficacy of supplemented fat-soluble vitamins [54]. Therefore, more mechanistic studies are required to elucidate the additional contribution to the efficacy of the MMNs by the functional fat fraction of the RUSF.

The study has a number of limitations, which need to be addressed. First, the projected sample size was not attained, limiting the study's statistical power. The main reason for this was the unpredictable security situation, which obliged field staff to restrict their field activities during the screening period. In addition, ACF-France decided to restrict the target sample population to households with high dependency ratio, which resulted in difficulties in achieving the anticipated sample size. It is noteworthy to mention nonetheless that the post hoc calculated intra-cluster correlation coefficient of 0.0075 showed a lower value than the anticipated value of 0.01 used for the sample size calculation. Second, the study participants were not blinded with respect to the intervention assignment because of the type of supplement (paste) provided to children. We opted for a cluster-randomized design to minimize the effects of contamination (RUSF sharing); however, clusters were not always geographically separated from each other. Significantly fewer children from the control group were present at follow-up sessions; this was likely due to the following factors. The monthly food rations for both control and intervention participants were distributed to mothers/caretakers regardless of whether their child was present at the anthropometry sessions. Conversely, the RUSF was given only if the child was present at the anthropometry sessions. This factor, while limiting follow-up observations of children in the control group, also ensured that households received monthly food rations regardless of their participation in the study, thereby sustaining their exposure to the control treatment. Finally, child morbidity was recorded through caretaker recall, which could have resulted in underestimation.

In conclusion, adding child-targeted RUSF supplementation to a general food distribution resulted in increased hemoglobin status and linear growth, accompanied by a reduction in diarrhea and fever episodes. However, we could not find clear evidence that adding RUSF to a household food ration distribution of staple foods was more effective in preventing acute malnutrition. Other context-specific alternatives for preventing acute malnutrition should therefore be investigated.

## Supporting Information

Text S1 **Study protocol.**
(PDF)

Text S2 **CONSORT checklist.**
(DOC)

## Abbreviations

ACF-France: Action Contre la Faim–France
HAZ: height-for-age Z-score
LNS: lipid-based nutrient supplement
MMN: multiple micronutrient
MUAC: mid-upper arm circumference
NCHS: National Center for Health Statistics
OR: odds ratio
RUSF: ready-to-use supplementary food
RUTF: ready-to-use therapeutic food
SD: standard deviation
SES: socioeconomic status
WHO: World Health Organization
WHZ: weight-for-height Z-score
