# Supplementary material for: The Effect of Adding Ready-to-Use Supplementary Food to a General Food Distribution on Child Nutritional Status and Morbidity: A Cluster-Randomized Controlled Trial
Source: PLoS Med. 2012 Sep 18;9(9):e1001313. doi: 10.1371/journal.pmed.1001313 (PMC3445445; doi:10.1371/journal.pmed.1001313)
Supplement: Text S1 — Study protocol. (PDF) [file pmed.1001313.s001.pdf]

UGENT0410

Research protocol

PREVENTING ACUTE MALNUTRITION IN 6-36 MONTHS OLD CHILDRENBY PREVENTIVE READY-TO-USE-FOOD (RUSF) SUPPLEMENTATION IN URBAN CHAD: THE PREAMA PROJECT

Action Contre la Faim (France)& Ghent University (Belgium) in collaboration withWFP

**Sponsor and operational executing agency:**	**Action Contre la Faim France<br>Rue Niepce 4<br>75662 PARIS Cedex 14B<br>France**
**Scientific executing agency:**	**Department of Food Safety and Food Quality<br>Ghent University<br>Coupure Links 653<br>9000 Gent - Belgium**

The PREAMA project

Summary sheet

**Country:**	Chad
**Type of project:**	Research project<br>Cluster-randomized trial
**Project Title:**	The PREAMA project. Effectiveness and cost-effectiveness of a Ready-to-Use-Food (RUSF) Supplement to prevent acute malnutrition in children between 6-36 months in urban Chad.
**Version:**	Version 2.0, dated 01-APRIL-2010
**Project codes:**	UGENT0410<br>ClinTrials.Gov NCT01154595
**Project summary:**	The overall objective of this project is to assess the effectiveness and cost-effectiveness of a Ready-to-Use Supplementary Food supplement in the prevention of moderate acute malnutrition in children 6-36 months old. The evaluation will be based on a cluster-randomized trial including 14 geographic clusters in the city of Abéché, Chad. 2000 children will participate. The main outcomes are:<br>- Wasting incidence rate over 4 months (Weight-for-Height <-80% of NCHS or presence of bilateral pitting oedema)<br>- Morbidity (Acute Respiratory Infections, diarrhoea, anaemia and malaria incidence) during the project period<br>- Cost-effectiveness (ICER, incremental cost-effectiveness ratios)
**Project Duration:**	11 months
**Starting Date:**	May 2010
**Completion Date:**	November 2011
**Executing Agency:**	Action Contre la Faim (France) & Ghent University
**Sponsor :**	Action Contre la Faim (France)
**Scientific Agency:**	Ghent University
**Project Contacts:**	- Patrick Kolsteren (MD, PhD), professor Ghent University Patrick.Kolsteren@ugent.be<br>- LievenHuybregts (PhD), Post-doctoral researcher Ghent University Lieven.Huybregts@UGent.be<br>- Myriam Ait Aissa (MSc), responsible research Action Contre la

Faim France, Maitaissa@actioncontrelafaim.org

Project Budget:

Budget Item	Cost (EURO)
INVESTMENT COSTS	26.097,00
OPERATING COSTS	143.151,00
SERVICE CONTRACTS / STAFF	466.916,00
INTERNATIONAL TRAVEL EXPENSES	56.020,00
SHIPMENT COSTS	74.736,00
**TOTAL**	**766.920,00**

STATEMENT OF COMPLIANCE & CONFIDENTIALITY

By signing this protocol, the Principal Investigator and the Sponsor acknowledge and agree:

This protocol contains all necessary information for conducting this study. The Principal Investigator commits to supervise that study is conducted in compliance with the protocol, amendments, applicable procedures and other study-related documents provided by the Sponsor, and in compliance with applicable ethical, GCP and regulatory requirements of Chad.

The protocol and all relevant information which was provided by the Sponsor will be made available to all physicians, nurses and other personnel who participate in conducting this study. The Investigator will use this material for their training so that they are fully informed regarding the objectives and the conduct of the study.

This document may contain information that is privileged or confidential. As such, it may not be disclosed to any other than involved research staff, the concerned Ethics Committee(s), unless specific permission is granted in writing by Action Contre la Faim France, or such disclosure is required by federal or other laws or regulations. In any event, persons to whom the information is disclosed must be informed that the information is privileged and confidential and may not be further disclosed by them.

The Sponsor of this study – Action Contre La Faim France – and the scientific executing agency – Ghent University - will at any time have access to the source documents from which Case Report Form information may have been generated. The Case Report Forms and any other data pertinent to this study are the property of the Sponsor, who may utilize the data in various ways, as agreed with the research partners.

COORDINATING INVESTIGATOR ACTION CONTRE LA FAIM FRANCE

Title, Name: Myriam Ait-Aissa, MSc Date: ________________

Signed:

DIRECTOR OF ACTION CONTRE LA FAIM FRANCE:

Title, Name: François Danel, Executive director Date: ________________

Signed:

PRINCIPAL INVESTIGATOR:

Title, Name: Prof dr Patrick Kolsteren Date: ________________

Signed:

ASSOCIATED INVESTIGATOR GHENT UNIVERSITY:

Title, Name: Dr Lieven Huybregts Date: ________________

Signed:

Signing this document, I commit to carry out the trial according to the protocol and to all the applicable ethical and regulatory requirements. I also acknowledge the paragraph relevant to study confidentiality and authorize both Ghent University, Belgium and Action Contre La Faim France to record my data on a computerized archive containing all the data pertinent to the study.

GLOSSARY

**ACF**	Action Contre La Faim
**CMAM**	Community-based Management of Acute Malnutrition
**CNNTA**	Centre National de Nutrition et Technologie Alimentaire
**CSB**	Corn-Soy Blend
**DHS**	Demographic Health Survey
**GCP**	Good Clinical Practice
**HAZ**	Height-for-Age Z score
**HH**	Household
**ICER**	Incremental Cost-Effectiveness Ratio
**LMI**	Low- and Middle Income countries
**MAM**	Moderate Acute Malnutrition
**MSF**	Médecins Sans Frontières (Doctors Without Borders)
**MUAC**	Mid-Upper Arm Circumference
**NCHS**	National Centre for Health Statistics USA
**PI**	Principal Investigator
**RUSF**	Ready-to-Use Food
**RUSF**	Ready-to-Use Supplementary Food
**RUTF**	Ready-to-Use Therapeutic Food
**SAM**	Severe Acute Malnutrition
**UNICEF**	United Nations Children's Fund
**UNSCN**	United Nations Standing Committee on Nutrition
**WFP**	World Food Program
**WHO**	World Health Organization
**WH**	Weight-for-Height
**WHZ**	Weight-for-Height Z score

1. Background

1.1 Justification of the problem

Acute malnutrition is a major contributor to under-5 mortality and morbidity in low and middle income (LMI) countries (1) Clinical guidelines for the management of severe acute malnutrition (SAM; WHZ≤-3 or presence of bilateral pitting oedema) have been available and their implementation has yielded satisfying results(2;3). In addition, outpatient community-based therapeutic programs for uncomplicated SAM infants using a peanut-based spread fortified with micronutrients, also known as ready-to-use therapeutic food (RUTF) showed increased coverage and recovery rates>75% in resource poor settings (2;4).

In comparison to SAM, research on the treatment of Moderate Acute Malnutrition (MAM; WHZ has started more recently with a more formal interest of WHO, WFP, UNICEF (5). Supplementary feeding programs by national and international organizations to treat MAM (-3≤WHZ<-2) use often a blended flour of cereals and pulses, albeit fortified with micronutrients (6). More recently, studies have demonstrated that using ready-to-use fortified supplementary spreads (RUSF) to treat moderately malnourished and underweight children was more efficacious on weight gain than using blended flours (7;8;8).

Recently, the management of acute malnutrition has shifted from a recuperative model to a more preventive one. One of the reasons is that the individual nutritional rehabilitation of malnourished children using special fortified spreads is quite expensive. Secondly, a programmatic study involving blanket food assistance to tackle child undernutrition was shown more effective than food assistance programs targeted at undernourished children (9).In a cluster randomized trial in rural Niger, Isanaka and colleagues (10) evaluated the preventive effect of a blanket distribution of 92g RUTF in children aged 6-60 months during the yearly hunger gap which resulted in a significant decline in wasting and severe wasting incidence over a period of 8 months. However, it remains questionable if the use of a therapeutic dose of RUTF to prevent malnutrition is cost-effective and a feasible strategy. In addition, there exists the risk that such high dose for the youngest children (for example between 6 and 12 months) could substitute for breastfeeding. A second study in rural Niger by Defourny and colleagues(11) evaluated the preventive effect of a smaller dose of fortified spread (45g), called Ready-to-Use Supplementary Food (RUSF) specifically designed for the prevention of malnutrition. This study provided a six-month supplementation with RUSF in children between 6 and 36 months old and showed that the previously noticed seasonal rise in admissions to nutritional rehabilitation programs during the *hunger gap* noticed in the previous years, was flattened. However, the study did not include a parallel control group, so it cannot be excluded that time-contextual factors over the years could have influenced the results. In addition, Galpin and colleagues showed in a study using stable isotopes that children (AGE) receiving such dose of fortified spread did not lead to lower breastmilk intake compared to more conventional porridge as complementary food(12).

1.2 Urban Chad - Abéché

The republic of Chad is a landlocked country in central Africa bordered by Lybia, Sudan, Cameroon, Central African Republic, Nigeria and Niger. The city of Abéché is the capital of the Eastern Ouaddaïregio, near the border with Sudan (**Figure 1**) The city is divided in six administrative districts consisting of in total 48 sectors. Every sector is subdivided in a number of administrative subsectors.

The East of Chad is characterized by a semi-arid climate, which can be divided in a hot dry period (December-June) and a rainy season (July-October/November). A hunger gap with relatively increased food insecurity can be identified during May-October. Abéché and its surroundings have suffered from the neighboring civil war in Darfur (Sudan) between 2007-2008, which resulted in an influx of refugees camped around the city.
In Chad, no less than 13.5% of the under-5 children suffer from acute malnutrition or wasting (13). In 2007, Chad had the 3rd highest under-5 child mortality rate (209 per 1000 live births) of the world(14).

Recently, Action Contre la Faim France conducted two nutritional surveys in representative samples of the population of Abéché. A first survey[1] in 853 children aged 0-59 months, conducted at the beginning of the rainy season (June 2009), reported a wasting (WHZ<-2 of the NCHS reference) prevalence of 20,6% in under-five children with 3,2% severe wasting. In addition, a stunting (HAZ<-2 of the NCHS reference) prevalence of 26,3% was found with a severe stunting prevalence as high as 10,0%. A second survey[2] in 650 under-five children was conducted during the post-harvest period (January 2010). This survey showed a wasting prevalence (or Global Acute Malnutrition (GAM) prevalence) of 16,8% (severe wasting prevalence at 2,0%). The wasting (or GAM) prevalence in both of the previously mentioned surveys was above the cut-off of 15% which describes the overall nutritional situation as 'critical'.
Both studies demonstrated that exclusive breastfeeding practices during the first 6 months were virtually absent (prevalence < 3%), which was also confirmed by the last national DHS of 2004 (13).

---

[1] Action Contre La Faim. Enquête Nutritionnelle anthropométrique et de Mortalité Abéché Ville, Tchad. 2009, Internal report

[2] Action Contre La Faim. Enquête Anthropométrique nutritionelle et de mortalité retrospective en Abéché ville, Tchad. 2010, Internal report

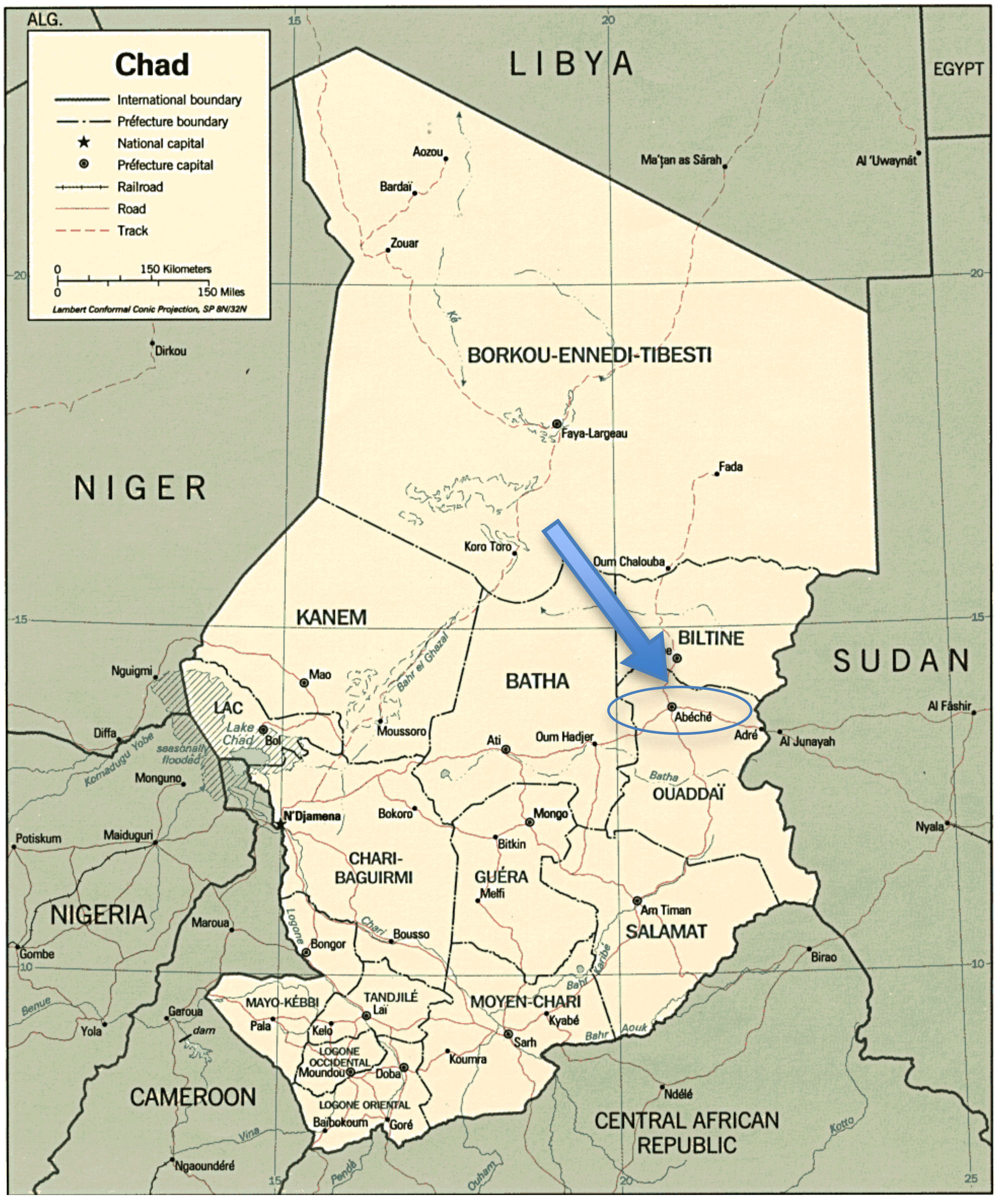

Figure 1: Situation of Chad and the city of Abéché

The seasonality of the incidence of child acute malnutrition is illustrated by **Figure 2**, which shows the number of child/patient entries in health facilities for nutritional rehabilitation. This period coincides with the so-called *hunger gap* (May-October), which is characterized by food insecurity, higher food prices and an increased infection load due to the rainy season.

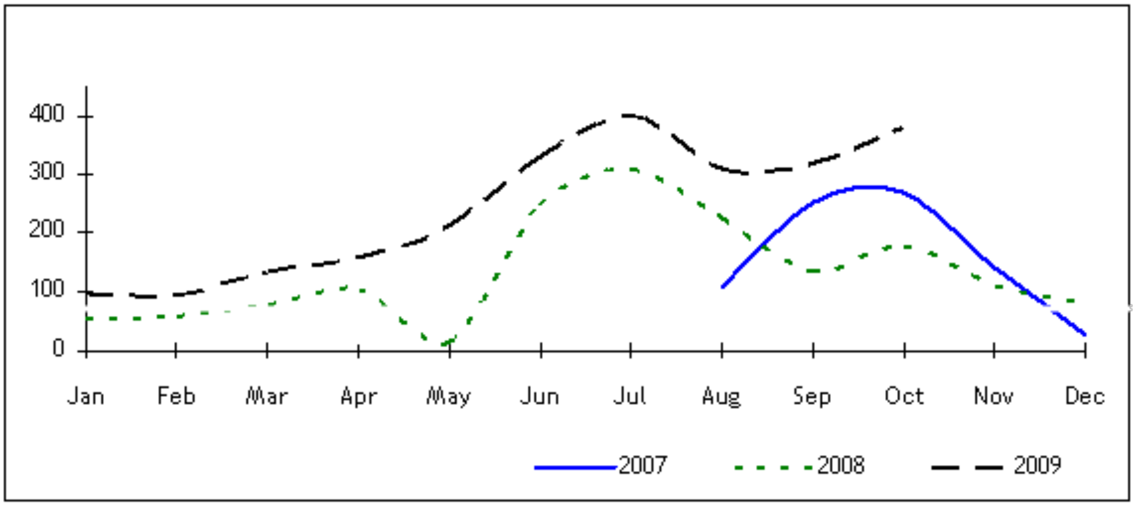

Figure 2: Patient/child entries in nutritional rehabilitation facilities in Abéché for the period 2007-2009 (source: ACF)

The study will be conducted in twelve subsectors of the city of Abéché, corresponding with seven administrative sectors that were identified as the most vulnerable in terms of food security and sanitation of the entire city. All households that are included in the study will receive food rations, which are provided conditional on the attendance of child's caretaker in a number of sensitization sessions on hygiene promotion, sanitation and dietary counseling concerning optimal complementary feeding and breastfeeding practices. Children of half of the clusters will receive monthly, an additional unconditional ration of RUSF (Plumpy Doz®) as a supplementary nutritional support to prevent child malnutrition related to the seasonal *hunger gap*. Worldwide, the window of opportunity to conduct complementary feeding interventions to prevent acute malnutrition is situated between 6 and 24 months (15). For this study the age range is extended up to 36 months, given the lack of child growth data for this specific population.

As previously mentioned, fortified energy dense spreads have shown their efficacy and effectiveness as RUTF and RUSF to treat SAM and MAM respectively. In addition, preventive RUTF is a valuable, though costly, instrument in the fight against malnutrition. However, strong evidence is lacking that a small portion of RUSF can prevent child malnutrition in a (cost)-effective way. More importantly, it remains to be evaluated what the contribution is of such a specifically designed food supplement like RUSF, embedded in a food assistance program aimed at preventing food insecurity and child malnutrition. Ideally not only effectiveness, but also relative cost-effectiveness of such combined intervention should be evaluated.
In addition, almost all available studies involving the use of fortified spreads in the treatment or prevention of malnutrition were conducted in rural areas. Programmatic studies involving the use of a RUSF component in an urban area are therefore warranted.

a. General Objective of the Project

The overall objective of this project is to assess the effectiveness, cost-effectiveness of RUSF to prevent acute malnutrition or wasting (WH<80% of the median of NCHS reference or presence

of bilateral pitting oedema) in children aged 6-36 months if incorporated in a program of conditional household food assistance. The amount of monthly family food ration that is provided to the household is conditional on the presence of household members during specific sensitization sessions involving hygiene promotion, sanitation and dietary counselling concerning complementary feeding and breastfeeding practices.

b. Specific Objectives of the Project

1. To assess the acceptability of RUSF by the children aged 6-36 months and by their caretakers

2. To assess the effectiveness of RUSF in preventing child acute malnutrition (wasting) as a component in a preventive intervention package to reinforce household food security, hygiene, sanitation and child feeding practices.

3. To assess the cost-effectiveness of RUSF in preventing child malnutrition (wasting) as component in a preventive intervention package to reinforce household food security, hygiene, sanitation and child feeding practices.

c. Hypotheses

1. The provided dose of 46g RUSF is well-accepted and consumed by children (6-36 months).

2. Caretakers show a positive attitude towards preventive RUSF.

3. Providing additional RUSF in a preventive intervention package to reinforce household food security is more effective and cost-effective for preventing child malnutrition compared to providing this intervention package without RUSF.

It is also expected that screening the under-3y population will enhance the awareness of the families and will have beneficial effects at the population level. Therefore, such strategy could have beneficial effects not only on participating children, but also at the meso-level in this urban area.

2. Research Methods

2.1Study population

The study will be conducted in the seven most deprived sectors of the city of Abéché. The selection of these 7 sectors (Hilekinine, Dabanaïr, Hilélé,Salamat I et II, Djinene Truck and

Djinene Fock) was done using a community participatory approach and by using a propensity score based on a set of criteria: water availability, job opportunities, public sanitation, socio-economical character, water availability, education quality and number of children referred to health center. According to estimations of the last nutritional survey in 2010 conducted by ACF, these 7 sectors represent ± 4000 households and approximately 2800 children aged 6-36 months.

2.2 Study interventions

a. Settings and Programmatic conditions

This study is embedded in an intervention program of conditional food distribution in seven vulnerable sectors of the city of Abéché. The main goal of this program is to reinforce household food security in the most vulnerable households of Abéché by preventive conditional food transfers. Food distribution will be organized conditional on the attendance of child's caretakers to sensitization sessions involving hygiene promotion, sanitation and dietary counseling concerning complementary feeding and breastfeeding practices. In addition participants will be asked to participate into a community mobilization program.

Household food rations

All households enrolled in the study will receive blanket food rations. This food package will consist of a fixed monthly ration of sorghum, sugar, vegetable oil and iodised salt (**Table 1**) provided in kind by WFP. To facilitate the distribution and because it is difficult to determine the exact size of household, all households will receive the same amount of family rations irrespective of the household's size. A maximum of 20 daily rations per month is foreseen for every household. The mean theoretical energy support from the provided food is calculated to be 1800 kcal.person-1.day-1, covering approximately 86% of the daily energetic requirements for a population at risk of an emergency (17). The number of food rations to be distributed per household will be proportional to its size: 1.5 rations for household of 1 to 2 members; 3 rations for a household of 3 to 4 members; 5 rations for households of 5 to 6 members; 8 rations for households of 7 to 9 members and 11 rations for households of at least 10 members.

Table 1: Composition of a daily food ration per person as proposed by WFP

**Food**	**Quantity (g/person/day)**
Sorghum	425
Dried Beans	25
Vegetable oil	25
Sugar	20
Iodised salt	5
Total weight	500

Sensitization component

The sensitization program will consist of a number of workshops treating various themes on household water management, hygiene promotion, sanitation and dietary practices for children. In addition, participants will be asked to actively sensitize their neighborhood with the newly obtained knowledge and practices in a program of community mobilization.

A first component of the sensitization entails dietary counseling regarding optimal complementary feeding and breastfeeding practices based on the "Manual on Counselling the Mother" (18).The dietary recommendations will be consistent with those formulated in the WHO Guiding Principles for Complementary Feeding of the Breastfed Child (19) and the WHO Guiding Principles for Feeding Non-Breastfed Children 6-24 Months of Age (20). During these sessions caretakers will also be explained how to use the food rations in the preparation of complementary foods.
A second component involves various themes:

- Hygiene promotion (personal hygiene, hygiene on household level)
- Water quality (use of clean recipients, disinfection techniques for water, transport and storage practices)
- Sanitation (maintenance and utilization of latrines, waste water drainage,
- Food safety (Storage and preparation practices, pest management)
- Preventive health care (prevention of disease vectors,

A total of 5 days per month are scheduled for these workshops. In addition, during 11 days per month participants are asked to sensitize at least 3 different persons in their direct neighborhood. For this purpose participants will be asked to organize small group discussions to discuss the same themes as treated during the sensitization sessions.

On a community level additional communication strategies like street theater, posters, radio, etc will be used to support the efforts of the sensitization program.

The conditional character of the food distribution

Participants will be encouraged to participate into the sensitization program. A maximum of 20 food rations are scheduled per month. In function of the attendance pattern of the participants in these sessions, the monthly quantity of food rations for each household will be adjusted. A tolerance of 2 days of absence will be allowed. If a participant is absent for more than 2 days the number of daily food rations provided will decrease linearly in function of additional days of absence, eg. if a participant is absent during 3 days he/she will receive 1 food ration less per month.

b. Ready-to-Use Supplementary Food (RUSF)

RUSF is a specifically formulated food branded Plumpy Doz® and is produced industrially by Nutriset® (Malauny,France). RUSF is a powder-in-fat matrix fortified with multiple micronutrients. The RUSF spread consists of peanut butter, vegetable oil, maltodextrine, milk powder and a premixed cocktail of multiple micronutrients. **Table 2** shows the nutritional composition. RUSF spreads contain very little water (<2%) which guarantees a prolonged shelf life because of microbial stability. Plumpy Doz® was previously used in a program by MSF in Niger involving approximately 60 000 children aged 6-60 months to prevent child acute malnutrition (11).

Blanket distribution of RUSF will be organized on monthly basis. RUSF will be packed in plastic pots of 325g (4 units per month). The caretaker of the child will be advised to give the child three table spoons of RUSF (45g), one in the morning, one at noon and one in the evening. For this purpose plastic tablespoons will be provided to all participants. A daily doses of 45g RUSF will provide 250 kcal.
Caretakers will be explained that RUSF cannot replace breastfeeding and that breastfeeding should continue up to 24 months.

Table 2: Nutrient content of the daily ration of RUSF (PlumpyDoz®)

	RUSF
Energy (kcal)	247
Weight of daily ration (g)	46,3
Protein (g)	5,9
Lipids (g)	16,0
Vitamin A (µg)	400,00
Vitamin E (mg)	6,00
Thiamin (mg)	0,5
Niacin (mg)	6,0
Pantothenic Acid (mg)	2,0
Vitamin B-6 (mg)	0,5
Folic Acid (µg)	160
Vitamin B-12(µg)	0,9
Vitamin C (30mg)	30
Magnesium (mg)	60
Zinc (mg)	9,00
Iron (mg)	9,00
Copper (mg)	0,3
Potassium (mg)	310
Calcium (mg)	387
Phoshorus (mg)	275
Selenium (µg)	17
Manganese (mg)	0,17
Iodine (µg)	90,00

(source: Nutriset, Malauny, France)

2.3 RUSF acceptability study in children and caretakers

The acceptability of RUSF will be assessed on three levels. During a supervised feeding trial the acceptability of the RUSF by children will be assessed. A second phase will assess the acceptability of RUSF by the caretakers. Finally, an in-depth evaluation by focus group discussion will document various aspects of perceptions, attitudes and practices of RUSF by caretakers.

Phase 1: Supervised feeding study
The acceptability of RUSF by the children will be assessed during a supervised feeding trial. A convenience sample of 30 caretaker-child couples will be selected from the 7 sectors of the study area.

Inclusion criteria are:
- WH ≥ 80% of the median of NCHS reference
- Age between 7 and 36 months
- Currently being breastfed
- Already eating solid or semi-solid foods

Exclusion criteria are:
- Being under medical treatment
- Receiving nutritional supplements (eg. vitamins)
- Being malnourished (defined by WH<80% of the NCHS growth reference or the presence of bilateral pitting oedema)
- Presence of congenital malformations that can hamper food consumption
- Being allergic to one of the ingredients of RUSF (eg. milk or peanut protein)

The caretaker will be asked if the child has a history of peanut or milk allergy. All included children will receive a test quantity of 5g RUSF and will be monitored during 30 minutes if any signs of possible allergy occur (eg. skin itchiness).

Every caretaker will be offered a daily dose (45g) of Plumpy Doz® with a spoon to feed the child. Caretakers will be asked to monitor child's eating behavior. The initial and final weight of the RUSF will be recorded and subtracted to determine the consumed dose. The primary outcome will be the quantity RUSF the child is able to consume within a timeframe of 20 minutes. In addition, the time in which the recorded amount of RUSF is consumed, will be measured by field workers.

Phase 2: Caretaker's appreciation of RUSF
A second phase consists of a global appreciation of the attitudes of the caretaker concerning RUSF supplementation. A convenience sample stratified by study cluster (cfr 3.4) of 100 caretakers with children between 7 and 36 month will be given a two-weeks ration of RUSF to take home. Caretakers will be asked to feed their child three tablespoons of RUSF every day during three distinct moments during the day. After this evaluation period, caretakers will be invited for an interview. Caretakers will be asked to evaluate RUSF on odour, taste, flavour, aroma, colour, mouth feel, texture, consistency and total acceptability using a 5-point scale of

acceptability. In addition specific questions related to the caretaker's attitude towards RUSF will be asked using a semi-structured questionnaire.

Phase 3: In-depth evaluation of caretaker's attitudes towards RUSF
As a final phase, during the last month of the effectiveness study (cfr 3.4), in-depth focus group discussions will be conducted in order to identify caretakers' perceptions, attitudes and practices towards RUSF (Plumpy Doz®). This study is organised at the end of the effectiveness study to allow sufficient integration of the use of RUSF into the community. Four sessions of focus groups involving each 8 caretakers of not malnourished children will be organized. The gathered results will also provide additional insights in community acceptability of the RUSF supplementation program and can possibly enhance the interpretation of the obtained results of the effectiveness study (cfr 3.4)

2.4 Testing the effectiveness and cost-effectiveness of RUSF to prevent malnutrition in children aged 6 -36 months

Design

The study is designed as a two arm cluster randomised, controlled trial. Both arms participate in the conditional food transfer program of the project. One arm will receive additional RUSF in addition to this program.
The largest of 7 included sectors will subdivided in their administrative subsectors to obtain 14 clusters consisting out of 80-160 children aged 6-36 months. Every sector and subsector has a local community leader appointed by the city council of Abéché.

Study sample size

In order to detect a 50% reduction in the cumulative incidence of wasting (weight-for-height < 80% of the median NCHS growth reference or presence of edema) over a period of 4 months, with a statistical power of 80%, a type I error of 5%, an estimated mean cluster size of 100 children, and an intra-cluster correlation of 0.01, a sample size of 1220 children was calculated. Taking into account a study dropout of 15% a total sample size of 1435 children was projected.

Subject selection and randomization

Randomization of the 14 clusters between both intervention arms will be during a community ceremony. Small identical papers with the cluster names will be mixed in a bag and a volunteer will draw one paper at a time. The allocation will alternate between both study groups. The first draw will be allocated to the control group, the next to the intervention group, etc. Every cluster will dispose of a central distribution point where monthly distributions and child growth monitoring will take place.

A media campaign (street theatre, posters, tec.) in the 7 study sectors of Abéché will be organised to inform the population of the project. Subsector community leaders will be asked to inform their subsectors of the project. All children aged 6-36 months from vulnerable households will be invited to participate into the study. In the framework of the intervention program, vulnerable households are defined as households with a dependency ratio higher than 4. The dependency ratio is defined as the number of household members not in the labor force over those who are in the labour force. The purpose and modalities of the study will be explained to the community leaders and their informed consent will be sought.
During a six-days period all participants will be registered and screened. Written consent to participate in the study will be asked for every eligible child. If informed consent is obtained each eligible child will be included in the participants list and attributed an individual code.
Only eligible children that are presented at the registration session will be enrolled in the study.

Inclusion criteria will be:

- WH≥80% of median of NCHS reference without bilateral pitting oedema;
- Age ≥ 6 months and ≤36 months;
- Member of a household with a dependency ratio >4;
- Not planning to leave the study zone for the coming 4 months;

Exclusion criteria will be:

- Not showing appetite;
- Age < 6 months or age > 36 months
- Weight-for-Height % <80% and/or the presence of bilateral pitting oedema in children 6-36 months;
- Member of a household with a dependency ration ≤ 4;
- Clinical complications
- Presence of chronic illness, cardiac disease, congenital abnormalities, cancer;
- Being allergic to one of the ingredients of the RUSF.

If a child is detected to be MAM, it will be enrolled in a Community-based Management of Acute Malnutrition (CMAM) program that is operated by ACF (responsible MD Daraiya Alsimbilaya Idriss), in collaboration with UNICEF and the local health authorities. In case a child is found to be SAM, it will be referred to one of the CNS or city hospital for full medical diagnosis. In case it concerns a case of severe malnutrition without complications the child will be treated in ambulatory with RUTF according to the national protocol which prescribes a Community Therapeutic Care model as recommended by WHO/WFP/UNSCN/UNICEF (21). In the presence of clinical complications, children will be referred to the city hospital for appropriate in-patient management (3).

Intervention

Caretakers from clusters allocated to the RUSF arm will receive a monthly ration Plumpy Doz® (4 pots of 325g) in addition to the conditional food transfers. Caretakers will be advised to feed

their children Plumpy Doz ® (46 g d^{-1} or 3 tablespoons/day) during 3 different moments of the day.

Households in each of the clusters will be invited once a month to come to the distribution point. After notifying their presence on the registration list, child's anthropometry (weight, height/length, MUAC will be measured by two independent teams of 2 trained community health workers. Thereafter caretakers will receive a new monthly ration of RUSF together with a determined ration of the food package. The ration of distributed food depends on the number of training sessions the caretaker has attended to. Attendance lists of the sensitization sessions of the previous month will be consulted and the corresponding quantity of food ration will be calculated and distributed. To document compliance in case of RUSF, the empty plastic pots of the previous distribution will be recovered and counted at monthly distribution sessions.

Study outcomes

Main outcome measures will be:

- Cumulative wasting incidence over 4 months period (Weight-for-Height <80% of the median of NCHS) (main outcome);
- Cost-effectiveness.

Secondary outcome measures will be:

- Cumulative stunting incidence (Height-for-age <80% of NCHS reference);
- Haemoglobin concentration at endline or at program termination;
- Morbidity rate (ARI, diarrhoea, fever)
- Attitudes of caretakers towards RUSF;
- RUSF acceptability by children aged 6-36 months;
- Dietary intake from participating children

Measurements and procedures

Upon inclusion in the study, the caretaker will be interviewed to obtain baseline information including household composition, socio-economical status, dietary habits, child's age, breastfeeding practices and history of child and maternal illness.

<u>Anthropometry:</u>
Child's weight will be recorded using an electronic scale (SECA 834, Germany) or a hanging Salter scale (Avery Tronics, United Kingdom) to the nearest 100g. Length (≤ 24 months) and height (>24 months) will be recorded to the nearest 1 mm using wooden measuring board (Menuserie Besnard, France). MUAC will be recorded using non-stretchable plastic tape (Octopuss, France). All measurements will be taken in duplicate by 2 independent teams of trained staff in different rooms. Recorded measurements will be written down on separate forms to avoid that the second recording of anthropometry is influenced by the first. Staff that is

responsible for the recording of the anthropometrical measures will be blinded for the randomization.

Mortality and morbidity.
During the monthly contact moment trained community health workers will assess by interview disease episodes during the last week using a standardized questionnaire focussing on cough, short breathing, diarrhoea and fever episode. Diarrhoea episode is defined by at least 3 liquid stools per day. Fever In case of death, a verbal autopsy will be performed by a trained nurse or a physician using the standardized WHO questionnaire (22;23).

Haemoglobin concentration
The haemoglobin concentration will be measured at baseline and at the last distribution or when a child is discharged from the study by study nurses (eg when a child becomes malnourished). Finger blood haemoglobin concentration will be measured by spectrophotometry using a HemoCue device (HemoCueLtd, Dronfield, United Kingdom). The device will be calibrated on daily basis using a HemoCue Control Cuvette.

Post distribution monitoring
Every month a post distribution monitoring home visit will be organized in a subsample of 400 randomly selected households (stratified per study arm) to monitor the use of the provided food rations and RUSF. This survey will take place approximately 10 to 15 days following the distribution, and will cover questions on: appreciation of the food ration and RUSF, use of the food ration and RUSF (consumed, shared, exchanged, sold), food sources, income sources, repartition of expenditures at household level, and food intake at household level (quantity, quality, diversity).

Dietary assessment of children
In the months after the first and the last distribution an interactive quantitative 24-hr dietary recall will be conducted in a random subsample of 400 children (stratified per study arm) (24) Portion sizes will be estimated using household measures. Due to the lack of food composition table for Chad, the food composition table of neighboring Sudan will be used to convert the recorded food quantities into energy and nutrient intakes (25). In addition breastfeeding frequency will be estimated by recall.

Cost-effectiveness of interventions
For cost-effectiveness analysis, the net differential costs and key outcomes for each approach will be used to calculate the incremental cost-effectiveness ratios (ICER), i.e. the incremental costs divided by the incremental benefit. Specific cost-utility analysis will be performed to disentangle costs of a routine program including the intervention elements from those incurred from the research activities of the project.

Statistical analysis
EpiData version 3.1 (EpiData Association, Odense, Denmark) will be used for all data entry. Two data clerks will enter data in double. Data quality will be continuously evaluated through automatic consistency check.

All statistical analysis will be performed using STATA 11.0 (StataCorp, College Station, TX).. The effect of RUSF on the incidence of becoming malnourished (Weight-for-height <80% of NCHS or presence of bilateral oedema) over time will be assessed using mixed Poisson regression models. The intervention effect on continuous outcomes like WH%, HA%, muac will be assessed using linear mixed models with a random coefficient. All models will be adjusted for different levels of clustering: level-4: randomized clusters, level-3: households and level-2: children. Statistical significance is set at 5% for all analyses. All statistical tests will be two-sided.

Ethical consideration

Care will be taken to provide the best available treatment to address children needs. Children that suffer from acute malnutrition with medical complications will be referred to the nearest health centre or the city hospital for a full medical diagnosis by a medical doctor. Currently, local health services, with an institutional support by ACF, have implemented a Community Acute Malnutrition (CMAM) program that will include all study children that suffer from moderate acute malnutrition. In addition inpatient and outpatient treatment programs operated by the network of City Health Centres for severely acute malnourished (SAM) children are in place and dispose of sufficient material and capacity. At whatever level of management (community, health centre, reference hospital), good clinical practices will be applied and assessed throughout the study in both groups, e.g. vaccination and parasitic and infectious treatment will be provided when necessary. Caretakers will be explained the purpose of the study and invited to sign an informed consent form. Care will also be taken to ensure anonymity of participants during data management, data analysis and result dissemination.

An ethical clearance will be requested from the Ethics Committee of the University hospital of Ghent, Belgium. Chad does not have an officially appointed Ethics Committee; therefore the protocol will be evaluated by an *ad hoc* gathering of academic medical staff, representatives of the ministry of Health of Chad and the Centre National de Nutrition et Technologie Alimentaire (CNNTA).

3. PROJECT ADMINISTRATION PLAN

a. Project Management Structure:

ACF France will be the Sponsor and Executing Operational agency of the study. ACF is a non-governmental organization that has a longstanding experience in organizing the nutritional rehabilitation to malnourished children in various countries worldwide. ACF intervenes in food insecure populations and offers humanitarian aid in both emergency and non-emergency situations. ACF will be responsible for the overall coordination of the project and will provide all material and resources necessary for this study.

Ghent University is the Scientific Partner of the study. As such, it will guarantee the scientific aspects of the study under the form of the research protocol. In addition, multiple field visits will be undertaken to verify protocol adherence by the Executing Operational agency.

Scientifically and operationally, the responsibilities are shared as presented in **Figure 3**.

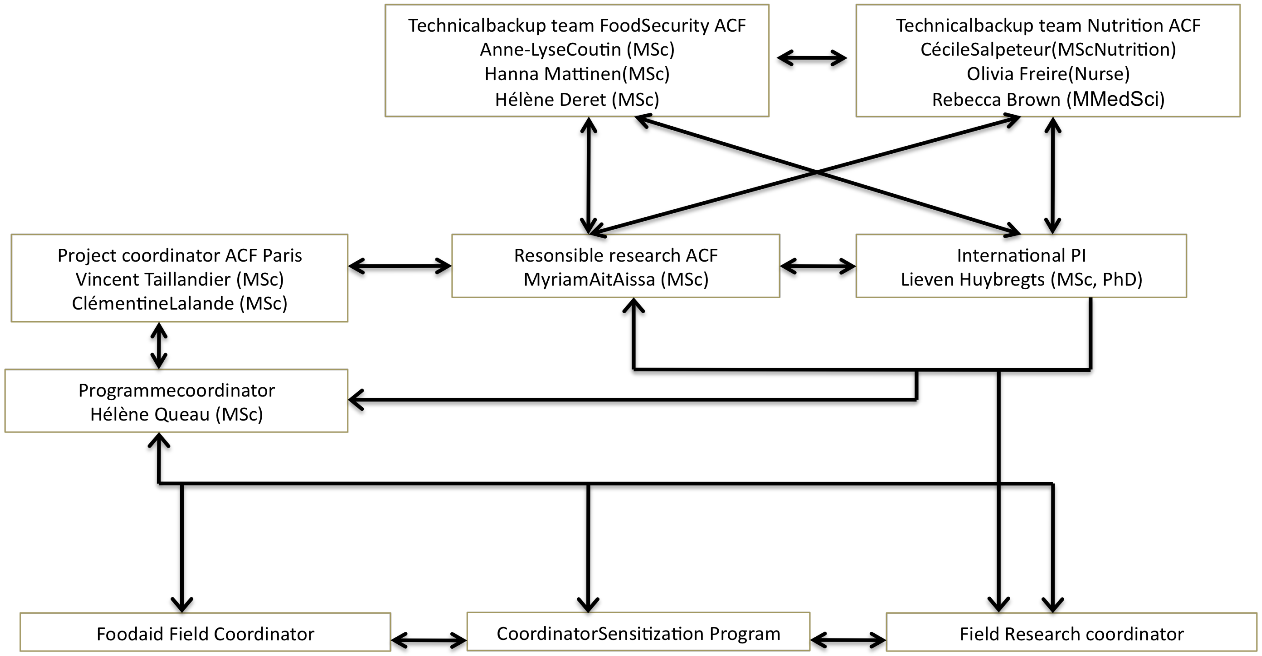

Figure 3: Main actors and responsibilities in the PREAMA project

b. Project Stakeholders

Project Stakeholders	Name	Role
Sponsor and Operational Executing Agency:	**ACF contact:**<br>Rue Niepce 4 - 75662 Paris Cedex 14<br>France Tel: +33.1.43.35.86.47<br><br>**Vincent Taillandier** (MSc) Project coordinator ACF France<br>vtaillandier@actioncontrelafaim.org<br><br>**Clémentine Lalande** (MSc) Project coordinator ACF France<br>clalande@actioncontrelafaim.org<br><br>**Hélène Queau** (MSc) Program Coordinator ACF Abéché<br>hqueau@actioncontrelafaim.org<br><br>**Cécile Salpeteur** (MSc Nutrition) HIV/AIDS & Nutrition advisor<br>csalperteur@actioncontrelafaim.org<br><br>**Myriam Ait Aissa** (MSc Nutrition) Responsible Research ACF<br>maitaissa@actioncontrelafaim.org<br><br>**Olivia Freire** (Nurse) Nutrition advisor ACF<br>ofreire@actioncontrelafaim.org<br><br>**Rebecca Brown** (MMedSci, MSc) Senior Nutrition advisor<br>rbrown@actioncontrelafaim.org<br><br>**Anne-Lyse Coutin** (MSc) Food Security advisor<br>alcoutin@actioncontrelafaim.org<br><br>**Hanna Mattinen** (MSc) Food security advisor<br>hmattinen@actioncontrelafaim.org<br><br>**Hélène Deret** (MSc) Senior Food Security advisor<br>hderet@actioncontrelafaim.org	- Coordination of research and logistics<br>- Financial management<br>- Supervision of research activities<br>- Quality control of data collection<br>- Dissemination of the results<br>- Clinical management of SAM and MAM

	**Daraiya Alsimbilaya Idriss** (MD)<br>Responsible clinical management SAM/MAM in Abéché<br>didriss@actioncontrelafaim.org	
Scientific Partner:	**Patrick Kolsteren** (prof, MD, PhD)<br>Department of Food Safety and Food Quality, Faculty of Bioscience Engineering, Ghent University<br>Coupure Links 653 – 9000 Gent<br>Patrick.Kolsteren@UGent.be<br>Tel: +32/9/2649377	- Principal investigator<br>-Design of the of study<br>-Quality control of the data collection<br>-Guarantor of research protocol<br>-Dissemination of the results
	**Lieven Huybregts**(MSc, PhD)<br>Department of Food Safety and Food Quality, Faculty of Bioscience Engineering, Ghent University<br>Coupure Links 653 – 9000 Gent<br>Lieven.Huybregts@UGent.be<br>Tel: +32/9/2649377	
Public Health partner	**Honoré Dembayo Djekaikoulayom (MD)**, Délégué Sanitaire Régional du Ouaddai,<br>hdembayo@yahoo.fr<br>Tel: + 235 6634 38 63<br>Tel: + 235 9914 39 10	- Clinical management of SAM and MAM<br>- Dissemination of study results
Other stakeholders	World Food Program	- offering foods for distribution

4. REFERENCES

(1) Black RE, Allen LH, Bhutta ZA, Caulfield LE, de Onis M, Ezzati M, et al. Maternal and child undernutrition 1 - Maternal and child undernutrition: global and regional exposures and health consequences. Lancet 2008 Jan 19;371(9608):243-60.

(2) Bhutta ZA, Ahmed T, Black RE, Cousens S, Dewey K, Giugliani E, et al. Maternal and Child Undernutrition 3 - What works? Interventions for maternal and child undernutrition and survival. Lancet 2008 Feb 2;371(9610):417-40.

(3) WHO. Management of severe malnutrition: a manual for physicians and other senior health workers. Geneva: WHO; 1999.

(4) Collins S, Dent N, Binns P, Bahwere P, Sadler K, Hallam A. Management of severe acute malnutrition in children. Lancet 2006 Dec 2;368(9551):1992-2000.

(5) Shoham J, Duffield A. Proceedings of the WHO, UNICEF, WFP and UNHCR Consultation on the Dietary Management of Moderate Malnutrition in Under-5 Children. Geneva: WHO; 2008.

(6) Chaparro CM, Dewey KG. Use of lipid-based nutrient supplements (LNS) to improve the nutrient adequacy of general food distribution rations for vulnerable sub-groups in emergency settings. Maternal and Child Nutrition 2010 Jan;6:1-69.

(7) Kuusipalo H, Maleta K, Briend A, Manary M, Ashorn P. Growth and change in blood haemoglobin concentration among underweight Malawian infants receiving fortified spreads for 12 weeks: A preliminary trial. Journal of Pediatric Gastroenterology and Nutrition 2006 Oct;43(4):525-32.

(8) Matilsky DK, Maleta K, Castleman T, Manary MJ. Supplementary Feeding with Fortified Spreads Results in Higher Recovery Rates Than with a Corn/Soy Blend in Moderately Wasted Children. Journal of Nutrition 2009 Apr;139(4):773-8.

(9) Ruel MT, Menon P, Habicht JP, Loechl C, Bergeron G, Pelto G, et al. Age-based preventive targeting of food assistance and behaviour change and communication for reduction of childhood undernutrition in Haiti: a cluster randomised trial. Lancet 2008 Feb 16;371(9612):588-95.

(10) Isanaka S, Nombela N, Djibo A, Poupard M, Van Beckhoven D, Gaboulaud V, et al. Effect of Preventive Supplementation With Ready-to-Use Therapeutic Food on the Nutritional Status, Mortality, and Morbidity of Children Aged 6 to 60 Months in Niger A Cluster Randomized Trial. Jama-Journal of the American Medical Association 2009 Jan 21;301(3):277-85.

(11) Defourny I, Minetti A, Harczi G, Doyon S, Shepherd S, Tectonidis M, et al. A Large-Scale Distribution of Milk-Based Fortified Spreads: Evidence for a New Approach in Regions with High Burden of Acute Malnutrition. Plos One 2009 May 6;4(5).

(12) Galpin L, Thakwalakwa C, Phuka J, Ashorn P, Maleta K, Wong WW, et al. Breast milk intake is not reduced more by the introduction of energy dense complementary food than by typical infant porridge. Journal of Nutrition 2007 Jul;137(7):1828-33.

(13) Ouagadjio, Bandoumal, Kostelngar N, Tchobkréo B, Riradjim M, Tokindang J, et al. Enquête Démographique et de Santé Tchad 2004. Calverton, MD: INSEED et ORC Macro (in French); 2004.

(14) UNICEF. The State of the World's Children 2009. New York: UNICEF; 2009.

(15) Victora CG, de Onis M, Hallal PC, Blossner M, Shrimpton R. Worldwide Timing of Growth Faltering: Revisiting Implications for Interventions. Pediatrics 2010 Mar;125(3):E473-E480.

(16) WHO Multicentre Growth Reference Study Group. WHO Child Growth Standards: Length/height-for-age, weight-for-age, weight-for-length, weight-for-height and body mass index-for-age: Methods and development. Geneva: WHO; 2006.

(17) UNCHR, WHO, UNICEF, WFP. Food and Nutrition Needs in Emergencies. 2004. 1-4-2010. Ref Type: Internet Communication

(18) WHO. Counsel the Mother. Geneva: WHO and UNICEF; 1997.

(19) PAHO, WHO. Guiding principles for complementary feeding of the breastfed child. Geneva: WHO; 2003.

(20) WHO. Guiding principles for feeding non-breast-fed children aged 6-24 months. Geneva: WHO; 2005.

(21) WHO, WFP, UNSCN, UNICEF. Community-based Management of Severe Acute Malnutrition. A joint statement by the World Health Organisation, the World Food Programme, the United Nations Standing Committee on Nutrittion and United Nations Children's Fund. Geneva: WHO/WFP/UNSCN/UNICEF; 2007.

(22) Coldham C, Ross D, Quigley M, Segura Z, Chandramohan D. Prospective validation of a standardized questionnaire for estimating childhood mortality and morbidity due to pneumonia and diarrhoea. Tropical Medicine & International Health 2000 Feb;5(2):134-44.

(23) Mobley CC, Boerma JT, Titus S, Lohrke B, Shangula K, Black RE. Validation study of a verbal autopsy method for causes of childhood mortality in Namibia. Journal of Tropical Pediatrics 1996 Dec;42(6):365-9.

(24) Gibson R, Ferguson E. An interactive 24-hour recall for assessing the adequacy of iron and zinc intakes in developing countries. Washington DC: ILSI; 2002.

(25) Boutros J. Sudan Food Composition Tables. Khartoum: Ministry of Health; 1986.
